# Making measuring family dinner quality more meaningful: Rescaling the ‘Family Dinner Index’ for ease of assessment and interpretation

**DOI:** 10.1016/j.mex.2025.103371

**Published:** 2025-05-15

**Authors:** Margie R. Skeer, Grace Hajinazarian, Rachael A. Sabelli, Sara C. Folta, Kendrin R. Sonneville, Misha Eliasziw

**Affiliations:** a Department of Public Health and Community Medicine, Tufts University, 136 Harrison Ave, Boston, MA, 02111, United States; b Friedman School of Nutrition Science and Policy, Tufts University, 150 Harrison Ave, Boston, MA, 02111, United States; c School of Public Health, University of Michigan, 1415 Washington Heights, Ann Arbor, MI, 48109, United States

**Keywords:** Measure, Methodology, Family dinner, Adolescent outcomes, Rescaling the Family Dinner Index from a continuous to an ordinal measure.

## Abstract

The ‘Family Dinner Index’ (FDI) is a tool that measures family dinner atmosphere and quality and includes parent (FDI-P) and child (FDI-C) versions. With this measure, higher-quality family dinners have been associated with reduced negative health- and risk-related outcomes among teens. This short report aimed to examine the utility of an ordinal version of the FDI to provide more meaning when using the measure. Using data from a national U.S. sample of 2,090 parent-teen dyads (10/2021–02/2022), the FDI measures were rescored and rescaled, and the associations with the same negative outcomes were tested and compared using the different versions. Both the continuous and ordinal versions had an approximate linear relationship between the FDI score and the log prevalence of each negative outcome. There were no significant differences across the estimates between the continuous and ordinal versions, demonstrating the utility of the rescored and rescaled FDI (both child and parent versions). Longitudinal research with the FDI is warranted to determine how dimensions of family dinners are associated with adolescent health and risk-related outcomes over time.

• The ‘Family Dinner Index’ (FDI) tool can be used as a continuous or ordinal measure of the quality of family mealtime interactions.
• The ordinal version of the FDI scale can provide researchers and practitioners with a more clinically relevant interpretation.

## Graphical abstract

**Figure:**
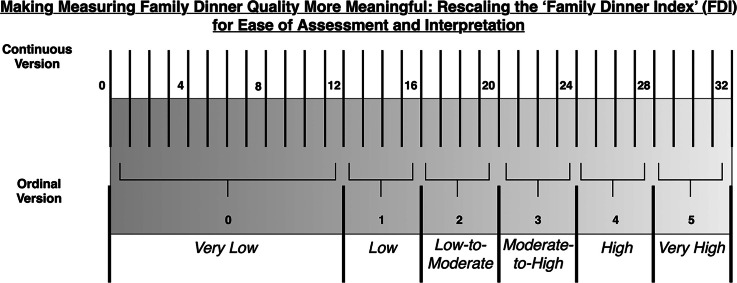

Specifications table

**Table:** 

Subject area:	Psychology
More specific subject area:	Substance Use
Name of your method:	Rescaling the Family Dinner Index from a continuous to an ordinal measure.
Name and reference of original method:	Skeer, M. R., Eliasziw, M., Sonneville, K. R., & Folta, S. C [1]. A new tool to capture dimensions of family dinners in relation to adolescent health and risk-related outcomes: The ‘Family Dinner Index’. *Preventive Medicine Reports, 35*, 102,318.
Resource availability:	Not applicable

## Background

The protective association between family meals and adolescent risk-related behaviors (substance use, disordered eating, and violence) has been consistently demonstrated across numerous literature and systematic reviews [[2], [3], [4], [5], [6]] and is considered to be conferred through enjoyment, communication, engagement, and connection at meals [5,7,8]. Since family meals have been consistently associated with a lower prevalence of risk-related behaviors, it has been considered an evidence-based parent-based preventive practice, and examples of studies which have demonstrated the effectiveness of family meals in this regard include The SUPPER Project [9], Project EAT [10], and the Family Meal Project [11]. However, a major gap in the literature has been that research in this area has primarily relied on family meal frequency in relation to these outcomes rather than what family interactions happen at the meal [2,3,5].

Recently, the ‘Family Dinner Index’ (FDI) was developed and validated, which is a new tool to measure family dinner atmosphere and quality; one for parents/guardians (termed ‘parents’ herein; FDI-P) and one for children (FDI-C). Based on qualitative research [7] and validity testing [1], the FDI is an original measure composed of eight items assessing four different constructs for parents and children (three overlapping and one distinct). These include (1) communication, (2) enjoyment, (3) digital distractions (which are reverse coded in analyses) for both parent and child versions, and (4) family bonding in the FDI-P and dinner logistics in the FDI-C [1]. The tool was validated across a national online sample of 2,090 parent-child dyads with youth aged 12–17 [1].

Total scores calculated for FDI-C and FDI-P range from 0 to 32, with higher scores indicating more positive family dinner atmospheres [1]. For the validation study, the scores were dichotomized at the median (≥21 for children and ≥24 for parents) [1]. The dichotomized FDI-C and FDI-P (‘high’ compared to ‘low’ FDI) measures were used to examine the relationship between family dinner quality and various health- and risk-related outcomes for youth. ‘High’ FDI scores were found to be associated with a reduced prevalence of four substance use indicators, violence, negative weight-related factors, not meeting daily fruit and vegetable consumption indicators and physical activity guidelines for youth, as well as with a reduced prevalence of an overall composite score of unhealthy behaviors [1].

### Study objective

For the validation study, the dichotomized measure was used because meaningful shifts in family interactions could not be observed for a one-point change in the FDI scores. However, dichotomizing FDI scores into ‘high’ and ‘low’ may not allow for a more nuanced understanding of differences in family meal quality levels. Therefore, the purpose of the present report is to propose a rescaled ordinal version of the FDI measure that would have a more meaningful interpretation of the quality of family meals and to extend its utility to researchers and practitioners.

## Method details

The constructs and items in the child version of the FDI-C included:

*Communication*

• “In general, how much do people talk to each other during family dinners?”
• “How much do you participate in the conversation during family dinners?”

*Enjoyment*

• “How much do you like being with your parent/guardian during family dinners?”
• “How much do you like being with members of your family during family dinners?”

*Digital Distractions*

• “How often are people allowed to talk, send messages, or watch something during family dinners using personal devices (for example, phones)?”
• “How often do people actively watch shows, movies, or sports games during family dinners (when it is not just on in the background)?”

*Dinner Logistics*

• “How much do you like chores that go along with dinners (for example, setting or clearing the table, washing the dishes) during family dinners?”
• “How much do you like helping with cooking during family dinners?”

The constructs and the items in the parent version of the FDI-P included:

*Communication*

• “In general, how much do people talk to each other during family dinners?”
• “How much does your child participate in the conversation during family dinners?”

*Enjoyment*

• “How much do you think your child enjoys family dinners in general (note: this does not include the food being served)?”
• “How much do you think your child likes having dinner with you?”

*Digital Distractions*

• “How often are people allowed to talk, send messages, or watch something during family dinners using personal devices (for example, phones)?”
• “How often do people actively watch shows, movies, or sports games during family dinners (when it is not just on in the background)?”

*Family Bonding*

• “How important is time to speak with your child about a reason for eating family dinners with your child?”
• “How important is building or maintaining stability in the family a reason for eating family dinners with your child?”

### From the original study

The original study provides more details on the development and validation processes, sample demographics, and results for the FDI measures [1]. Briefly, through an exploratory factor analysis with a loading factor greater than |0.5|, eight items were retained and grouped into the four FDI constructs. Stratified confirmatory factor analyses by the children’s age, gender, race, ethnicity, and region of residence in the United States were done to examine whether the FDI constructs persisted across different groups and found consistent results. These factor analysis methods have been used in public health and psychology research [[12], [13], [14]].

### For the current study

Each of the eight items was measured on a 5-point Likert scale (0: ‘Not at all’ to 4: ‘Very much’ for six items; and 0: ‘Never’ to 4: ‘Always’ for two items). The four constructs in the FDI measures each contain two items, so each construct represented eight points on the FDI scale with the total sum ranging from 0 to 32.

For the present report, analyses were first conducted using the continuous FDI version for each outcome to determine whether the associations were significant and were in the same direction as the dichotomized version from the original study [1]. Then, the continuous scores were rescaled and rescored to create an ordinal version, and analyses were conducted to determine how much they approximated the results using the continuous version.

With a range of 0–32, a score of 0 indicates that respondents ‘never’ or ‘not at all’ agree with all the items, and a score of 32 indicates that respondents ‘always’ or ‘very much’ agree with all the items. It was observed that a one-point change in FDI score did not have much meaning, whereas a four-point change was associated with a meaningful increase in family dinner quality. For example, a four-point increase, which corresponds with going from category 1 to 2 on the ordinal FDI scale would imply that an individual’s responses on average increased from being mostly 0′s and 1′s with some 2′s to mostly 2′s with some 1′s and 3’s. These scores were also used to ascribe labels to each point on the ordinal scale. Table 1 shows the full description of responses by the ordinal FDI categories.

**Table 1:** Description and distribution of the ordinal FDI version.

FDI Category	FDI Category Description	FDI-P ( %)	FDI-C ( %)
‘Very low’ (0)	Very low family dinner quality/atmosphere (e.g., mostly 0′s and 1′s, with some 2′s*).	1.7	5.7
‘Low’ (1)	Low family dinner quality/atmosphere (e.g., mostly 2′s with some 1′s and 3′s).	6.1	16.7
‘Low-to-moderate’ (2)	Low-to-mid-level family dinner quality/atmosphere (e.g., mostly 2′s and 3′s with some 1′s).	18.4	28.8
‘Moderate-to-high' (3)	High-to-mid-level family dinner quality/atmosphere (e.g., mostly 3′s and 4′s with some 2′s).	33.2	29.4
‘High’ (4)	High family dinner quality/atmosphere (e.g., mostly 4′s with some 2′s and 3′s).	28.8	14.6
‘Very high’ (5)	Very high family dinner quality/atmosphere (e.g., mostly 4′s with some 3′s).	11.9	4.9

⁎ Labels of FDI responses: '0': never/not at all; '1': rarely/a little; '2': sometimes/somewhat; '3': often/a lot; '4': always/very much.

In the process of rescaling and rescoring the FDI-C and FDI-P scores, very low frequencies were observed between 0 and 12 and, therefore, these scores were assigned to the ‘Very low’ category. Five more ordered categories were created corresponding to four-point increases. The following function describes the rescaling: x' = {0 if 0 ≤ *x* < 12; 1 if 12 ≤ *x* < 16; 2 if 16 ≤ *x* < 20; 3 if 20 ≤ *x* < 24; 4 if 24 ≤ *x* < 28; 5 if 28 ≤ *x* < 32}.

The results from the initial study [1] were replicated using generalized linear models (GLM) with a binomial distribution and log link. The results were presented as prevalence ratio (PR) estimates with corresponding 95 % confidence intervals (CI). Estimates were also examined for significant differences between the continuous and ordinal versions. Stata 18.0 was used to conduct all statistical analyses.

## Method validation

### Rescaling and rescoring the FDI measure from continuous to ordinal

When considered in the continuous version, the mean (SD; median; min-max) FDI-P score was 23.25 (4.54; 24; 6–32), and the mean FDI-C score was 20.31 (4.96; 20; 3–32). The categories constituting the ordinal FDI version are shown in Table 1, as well as the percentage of responses in each category.

### Comparing estimates between the continuous and ordinal FDI versions

There were no significant differences in the PR and 95 % CI estimates between the continuous and ordinal versions and the estimates were identical to the second decimal place (Table 2). Both versions had an approximately linear relationship between the FDI score and the log prevalence of the outcome.

**Table 2:** Adjusted prevalence ratios of unhealthy outcomes.

Outcomes	Ordinal FDI-C PR (95 % CI)	p-value		Ordinal FDI-P PR (95 % CI)	p-value
Substance use					
Ever had at least one drink of alcohol	0.88 (0.82, 0.93)	<0.001	0.90 (0.84, 0.95)	<0.001	
Ever tried cigarette smoking	0.91 (0.83, 1.00)	0.04	0.88 (0.80, 0.96)	0.004	
Ever used an electronic vapor product	0.93 (0.86, 0.99)	0.03	0.95 (0.88, 1.02)	0.18	
Ever used marijuana	0.86 (0.79, 0.92)	<0.001	0.88 (0.82, 0.95)	0.001	
**Violence**					
Ever been in physical fight	0.96 (0.91, 1.01)	0.10	0.98 (0.93, 1.04)	0.59	
**Weight perception**					
Describe oneself as overweight	0.92 (0.86, 0.98)	0.01	0.90 (0.84, 0.96)	0.001	
**Weight intention**					
Trying to lose weight	0.92 (0.85, 1.00)	0.045	0.92 (0.85, 0.99)	0.045	
**Health indicator not meeting guidelines**					
Fruit consumption	0.84 (0.81, 0.87)	<0.001	0.88 (0.85, 0.91)	<0.001	
Vegetable consumption	0.84 (0.81, 0.87)	<0.001	0.88 (0.85, 0.91)	<0.001	
Physical activity	0.93 (0.91, 0.94)	<0.001	0.93 (0.91, 0.94)	<0.001	

All estimates are from generalized linear models with log links and are adjusted for children’s age category, parents’ ethnicity and race, and census region.

Controlling for covariates, for the ordinal version, an increase in each FDI-C category (e.g. ‘very low’ to ‘low’; 'high’ to ‘very high’) was associated with reductions in the prevalence of each of the negative outcomes (PRs ranging from 0.84 to 0.93); and each increase in FDI-P-category was associated with reductions in the prevalence of each of the negative outcomes (PRs ranging from 0.88 to 0.93).

### Comparing the interpretation between the FDI versions

In a sample of 2,090 parent-child dyads, the significance of the estimates did not change when different FDI versions were used. However, meaningful decreases for negative child outcomes could not be interpreted with one-unit changes using the continuous FDI version, and more nuanced comparisons could not be made between ‘high’ and ‘low’ FDI scores using the dichotomized FDI version. Therefore, an ordinal version was developed. Both the continuous and ordinal versions had PRs in the same direction as the binary version, which supported the robustness of the FDI.

### Practical implications and use of the ordinal FDI version

The categories' labels help interpret more meaningful shifts in family interactions at meals in research and in practice with families. The labels and descriptions of family meal category levels will also allow for a better understanding of these category levels, and to identify which families fall into each group. For the clinical and practical use of the FDI measure, the ordinal version could provide an easier and more meaningful understanding of family meal quality. For research, the decision of which version to use will be based on different study questions and sample. Future research should focus on examining the FDI measure longitudinally to examine how the dimensions of family dinners are associated with adolescent outcomes over time. Furthermore, research which examines family meal-based interventions with potential public health implications can be more easily evaluated and disseminated using the ordinal version of the FDI measure.

## Limitations

Some important limitations to this study include the data being cross-sectional, limiting our ability to make temporal inferences about the associations observed. However, the practice of family meals is conceptually considered to begin early, and to precede the occurrence of risk-related behaviors in adolescents. The data were also collected via self-report, which may have resulted in some over-reporting for family dinner quality questions and under-reporting of risk-related behaviors [15]. Finally, the data were collected from 10/2021 to 02/2022, which overlapped with the COVID-19 pandemic. Therefore, mealtime patterns may not be generalizable to other contexts. Future longitudinal studies should consider re-assessing the criterion validity of the FDI measure with other samples.

## Ethics statements

This study involved human subjects, and informed consent was obtained from all participants before their involvement in the study. The Tufts University Social, Behavioral, Educational, and Research Institutional Review Board reviewed and approved the study protocol, and all procedures adhered to the ethical standards outlined in the Declaration of Helsinki. Participants were informed about the purpose of the study, their rights to withdraw at any time, and the confidentiality of their responses.

## CRediT authorship contribution statement

**Margie R. Skeer:** Conceptualization, Methodology, Investigation, Data curation, Validation, Writing – review & editing. **Grace Hajinazarian:** Methodology, Investigation, Data curation, Validation, Writing – review & editing, Writing – original draft. **Rachael A. Sabelli:** Investigation, Data curation, Writing – review & editing, Supervision. **Sara C. Folta:** Conceptualization, Methodology, Investigation, Data curation, Writing – review & editing. **Kendrin R. Sonneville:** Conceptualization, Methodology, Investigation, Data curation, Writing – review & editing. **Misha Eliasziw:** Conceptualization, Methodology, Investigation, Data curation, Writing – review & editing, Validation.

## Declaration of competing interest

The authors declare that they have no known competing financial interests or personal relationships that could have appeared to influence the work reported in this paper.

## Data Availability

Data will be made available on request.
